# Pulmonary tumor thrombotic microangiopathy and pulmonary veno-occlusive disease in a woman with cervical cancer treated with cediranib and durvalumab

**DOI:** 10.1186/s12890-018-0681-x

**Published:** 2018-07-11

**Authors:** Dante A. Suffredini, Jung-Min Lee, Cody J. Peer, Drew Pratt, David E. Kleiner, Jason M. Elinoff, Michael A. Solomon

**Affiliations:** 1 Critical Care Medicine Department, National Institutes of Health Clinical Center, Bethesda, MD USA; 2 Women’s Malignancies Branch, Center for Cancer Research, National Cancer Institute, National Institutes of Health, Bethesda, USA; 3 Clinical Pharmacology Program, Center for Cancer Research, National Cancer Institute, National Institutes of Health, Bethesda, USA; 4 Laboratory of Pathology, Center for Cancer Research, National Cancer Institute, National Institutes of Health, Bethesda, USA; 5 Cardiovascular Branch, National Heart, Lung, and Blood Institute, National Institutes of Health, Bethesda, USA

**Keywords:** Pulmonary tumor thrombotic microangiopathy, Pulmonary hypertension, Cediranib, Durvalumab, Cervical cancer

## Abstract

**Background:**

Pulmonary tumor thrombotic microangiopathy (PTTM) is a rare cause of pulmonary hypertension that is associated with malignancies and is marked by the presence of non-occlusive tumor emboli and fibrocellular intimal proliferation of small pulmonary arteries leading to increased pulmonary vascular resistance and right heart failure. The diagnosis of PTTM is challenging to make pre-mortem and guidelines on treatment are lacking.

**Case presentation:**

A 45-year-old woman with advanced squamous cell carcinoma of the cervix developed symptoms of dyspnea and evidence of right heart failure during a phase I clinical trial with cediranib and durvalumab. After an extensive evaluation, pre-capillary pulmonary hypertension was confirmed by right heart catheterization. Vasodilator therapy was initiated but resulted in the development of symptomatic hypoxemia and was discontinued. Despite continued supportive care, she continued to decline and was transitioned to hospice care. At autopsy, the cause of her right heart failure was found to be due to PTTM with features of pulmonary veno-occlusive disease (PVOD).

**Conclusion:**

PTTM and PVOD are important diagnoses to consider in patients with a malignancy and the development of right heart failure and may be manifestations of a spectrum of similar disease processes.

## Background

Pulmonary tumor thrombotic microangiopathy (PTTM) is a rare condition characterized by microscopic tumor cell emboli, which cause proliferative changes in the pulmonary microvasculature leading to a syndrome of hypoxemia, pulmonary hypertension, right heart failure and death [1]. In the initial report, unique pathologic changes in 21 patients were described with non-occluding microscopic tumor emboli limited to the small pulmonary arterial vessel wall, isolated or clumped in the vessel lumen and often with secondary thrombosis. The endothelial attachment of tumor cells was associated with fibrocellular intimal proliferation. The resultant obstruction of the small arteries and increase in pulmonary vascular resistance is thought to contribute to the clinical presentation of progressive cor pulmonale and death. It was notable that in nearly all the described cases lymphangiosis carcinomatosa was present, but the relationship to PTTM was unclear. Thus, PTTM is thought to be a unique clinical entity based on the presence of intimal proliferation, distinguishing it from obstructive pulmonary tumor emboli. In a larger case series, adenocarcinoma was the most common underlying malignancy and in nearly all cases the tumor emboli were positive for vascular endothelial growth factor (VEGF), platelet derived growth factor and tissue factor by immunohistochemistry [2]. Over-expression of these growth factors on the surface of the embolized tumor cell may result in a trophic effect on the pulmonary vascular endothelium, leading to the described pathologic findings [3]. Here we describe a woman being treated with the combination of a VEGF receptor (VEGFR) inhibitor and a programmed death-ligand 1 (PD-L1) inhibitor who developed pulmonary hypertension and right heart failure and was subsequently found to have PTTM with features of pulmonary veno-occlusive disease (PVOD).

## Case presentation

A 45-year-old woman with metastatic squamous cell carcinoma of the cervix refractory to standard of care chemotherapy was referred to the National Institutes of Health (NIH) for enrollment in a Phase I clinical trial (NCT02484404) of combination therapy with daily cediranib, a VEGFR tyrosine kinase inhibitor, and once every 2 weeks of durvalumab, a PD-L1 inhibitor. Her first four treatment cycles were well tolerated. Treatment related side effects included hypertension, subclinical hypothyroidism, non-nephrotic range proteinuria and mild diarrhea.

**Fig. 1:**
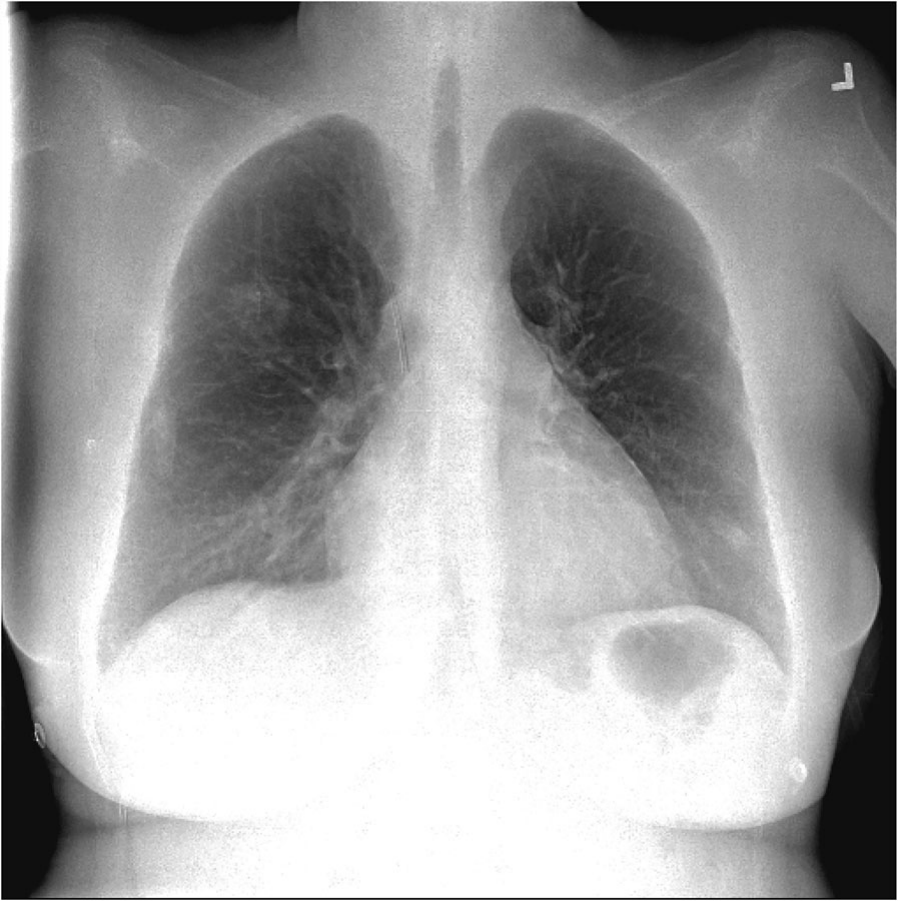
An anteroposterior chest radiograph demonstrating hazy bibasilar interstitial infiltrates. A port is noted as well in the right upper chest with catheter ending at the cavoatrial junction

**Fig. 2:**
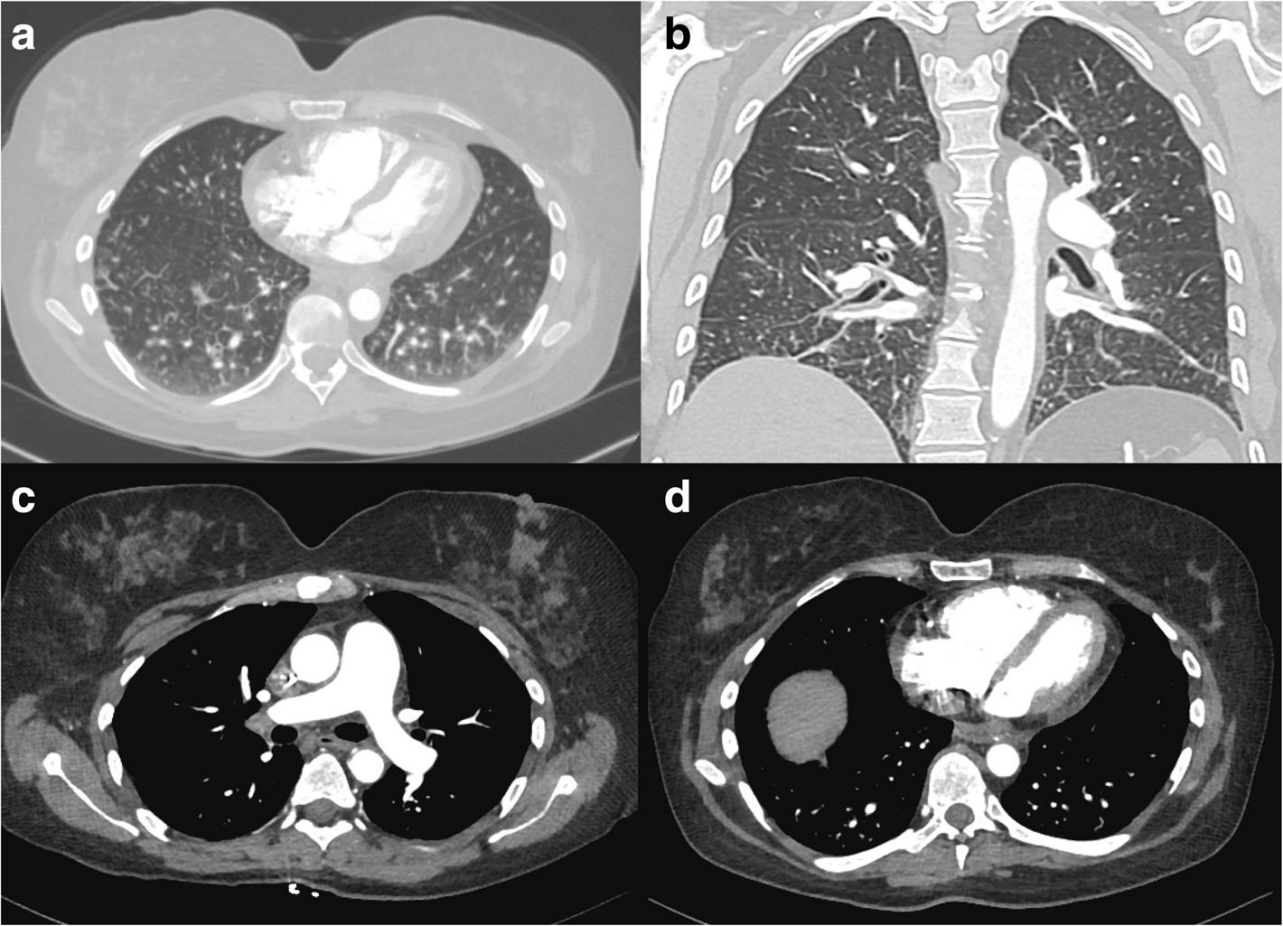
Contrast enhanced CT image with axial (**a**) and coronal (**b**) sections demonstrating a prominence of interstitial markings predominantly in posterior and basilar lung fields. In mediastinal windowing accentuating the pulmonary vasculature (**c**) the pulmonary artery trunk is enlarged and (**d**) the right ventricle and right atria appear larger in area than their corresponding left sided chambers; findings suggestive of pulmonary hypertension

**Fig. 3:**
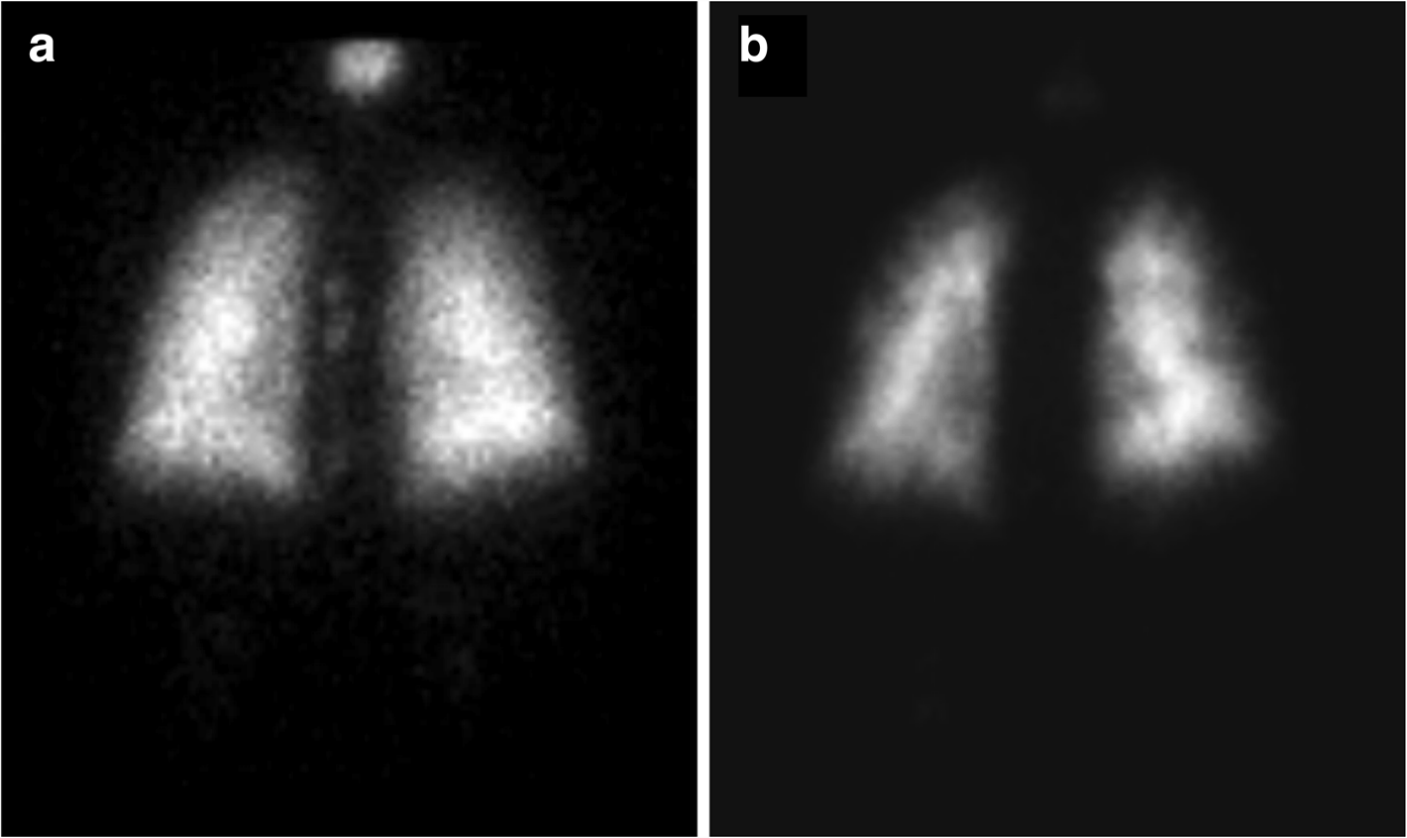
A posterior view of a ventilation (**a**) and perfusion (**b**) nuclear medicine scan demonstrate significant perfusion defects along the pleural margin (dark rim surrounding lung) that are not matched with a ventilation defect

During a routine study clinic visit the patient was found to be in sinus tachycardia. Upon further questioning, the patient noted progressive dyspnea on exertion and fatigue over the previous month and was therefore admitted to the NIH Clinical Center for further evaluation. Vital signs revealed a temperature of 37°C, heart rate of 120 beats per minute, a manual blood pressure of 90/72 mmHg without orthostatic changes and oxygen saturation ranging from 93 to 97% on room air. Physical examination findings were notable for a normal jugular venous pressure, regular heart rate without a prominent P2, clear breath sounds, and warm extremities without edema. Intravenous fluid was administered for possible dehydration due to diarrhea, but symptoms did not improve. A portable chest x-ray revealed hazy bibasilar interstitial markings (Fig. 1). Laboratory studies revealed a hemoglobin of 9.8 g/dL (normal range 11.2-15.7 g/dl), a platelet count of 159 k/μL (normal range 173-369 k/μL), normal coagulation indices (PT 13.8 s; aPTT 35.5 s; thrombin time 15.8 s), a D-dimer of 0.98 μg/mL (normal range 0.00–0.50 μg/mL), a fibrinogen of 517 mg/dL (normal range 177–466 mg/dL), a pro-brain natriuretic peptide of 4541 pg/mL (normal range 0–124 pg/mL) and a troponin-T of 0.022 ng/mL (normal range 0.000–0.009 ng/mL). Echocardiography demonstrated a dilated right ventricle with decreased function (tricuspid annular plane systolic excursion (TAPSE) of 8 mm; normal ≥17 mm) and an elevated right ventricular systolic pressure of 67 mmHg, new findings compared to her baseline echocardiogram completed just 4 months earlier. Cardiac MRI, performed to evaluate for possible treatment related myocarditis, demonstrated severely reduced right ventricular function (ejection fraction of 27%; normal 61±10%) with volume and pressure overload consistent with pulmonary hypertension, but no evidence of myocarditis. A CT angiogram showed no evidence of pulmonary emboli, however there was a prominence of interstitial markings (Fig. 2a, b), the main pulmonary artery was enlarged (Fig. 2c) and the right atrium and ventricle were both severely dilated (Fig. 2d). A ventilation (Fig. 3a) - perfusion (Fig. 3b) scan (VQ) demonstrated mismatched perfusion defects along the pleural margins that were interpreted as a high probability of pulmonary emboli. Doppler ultrasonography revealed no evidence of venous thrombosis in the lower extremities. Despite the equivocal findings, therapeutic anti-coagulation was started for possible pulmonary emboli.

Over the ensuing 10 days the patient’s clinical course progressively deteriorated heralded by a syncopal event and the development of significant resting hypoxemia. The patient was referred for right and left heart catheterization which confirmed pre-capillary pulmonary hypertension with an associated low cardiac output state (Table 1). Vasodilator challenge with nitric oxide (40 ppm) plus 100% oxygen paradoxically increased pulmonary artery mean and occlusion pressure, but did not result in symptomatic pulmonary edema. Balloon occlusion pulmonary venous blood sampling did not reveal any circulating tumor cells. Aggressive therapy for right heart failure was initiated including intravenous diuresis, inotropic support and pulmonary vasodilator therapy. Riociguat was initiated due to the concern for possible chronic thromboembolic pulmonary hypertension (CTEPH). Ultimately, intravenous epoprostenol was added due to persistent symptoms of severe right heart failure. Soon after starting epoprostenol the patient became severely hypoxic. As a result, pulmonary vasodilator therapy was discontinued and she was treated with diuretics and high flow oxygen. Given her advanced disease and poor functional status her protocol treatment was stopped and she was discharged home with hospice care. She died 1 day later and an autopsy was performed.

**Table 1:** Hemodynamic measures from left and right heart catheterization with vasodilator testing

	Room air	Nitric Oxide (40 ppm) plus 100% O_2_
Heart rate, beats per minute	107	105
Mean right atrial pressure, mmHg	18	14
Pulmonary artery pressure (mean), mmHg	72/30 (44)	72/34 (47)
Mean pulmonary artery occlusion pressure, mmHg	11	15
Aortic pressure (mean), mmHg	92/61 (72)	108/64 (80)
Left ventricle end-diastolic pressure, mmHg	10	–
Arterial blood gas, pH/pCO_2_/pO_2_	7.45/32/66	7.42/39/344
Arterial oxygen saturation, %	92.1	99.1
Mixed venous oxygen saturation, %	43.4	59.6
Cardiac index^a^, L/min/m^2^	1.5	1.6
Pulmonary vascular resistance, Wood units	12.4	11.1

^a^ Calculated by the Fick method using a measured hemoglobin of 11.1 g/dL and estimated VO_2_ (mL/min/m^2^)

**Fig. 4:**
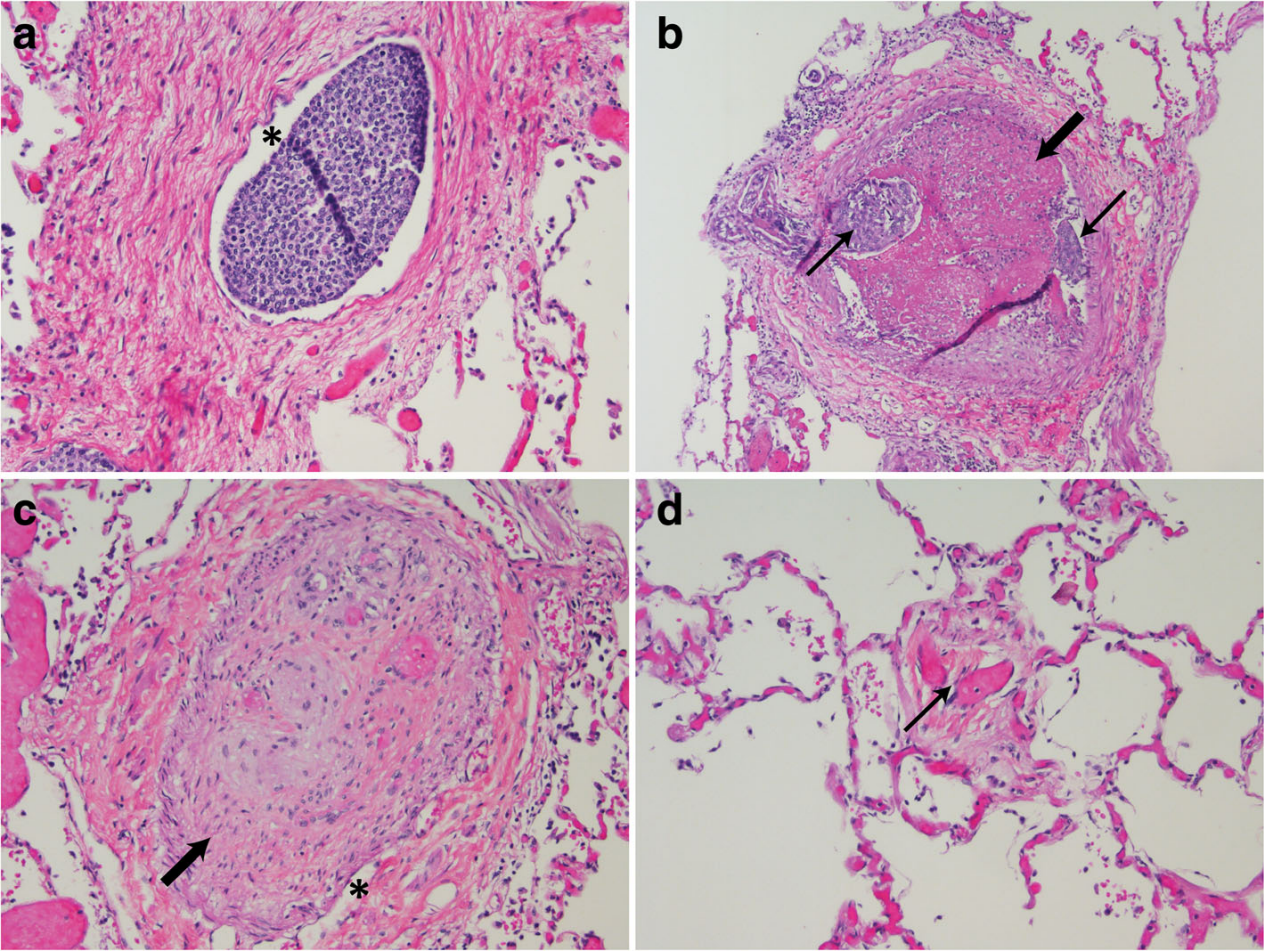
Pulmonary vascular disease associated with metastases. **a** Dilated lymphatic space (asterisk) containing tumor (200×). **b** Pulmonary artery with small tumor embolus (thin arrows) and both fresh and partially organized thrombus (thick arrow) (100×). **c** Pulmonary artery showing occlusion (thick arrow) and recanalization (asterisk) (200×). **d** Small pulmonary vein with narrowing and recanalization (thin black arrow) (200×)

Gross examination of the lungs showed scattered white plaques, nodular and gritty on palpation, covering the pleural surface of the lower lobes. Microscopic sections of the lung showed metastatic carcinoma with lymphangitic spread (Fig. 4a) within the septa and subpleural spaces with associated congestion and fibrosis. These findings were associated with fibromuscular thickening of lymphatic vessels which may represent a type of desmoplastic response to lymphatic obstruction. Pulmonary arterioles (Fig. 4b, c) were occluded by organized thrombi, some with evidence of recanalization, as well as smooth muscle proliferation and ingrowth of fibroblasts. Some arteries showed fresh thrombi with metastatic tumor foci. Pulmonary venules were also occluded with ingrowth of fibroblasts. Some of these vessels showed evidence of chronic occlusion and recanalization, (Fig. 4d) while others were completely occluded. No tumor cells were identified in any venules. No evidence of infection or pneumonia was identified. Collectively, these findings were most consistent with a primary diagnosis of PTTM with features of PVOD [1, 4].

## Discussion

We present a case of a woman with metastatic squamous cell carcinoma of the cervix who developed pulmonary hypertension and right heart failure during combination treatment with a VEGFR inhibitor and a PD-L1 inhibitor. At autopsy, she was found to have PTTM with features of PVOD.

A definitive pre-mortem diagnosis of PTTM is challenging as a number of other causes of right heart failure have a similar clinical presentation [5]. For example, our patient’s initial CT angiogram revealed prominent bibasilar septal lines, which may have represented lymphangitic spread of tumor, drug-induced pulmonary toxicity or PVOD [6]. Other non-specific radiographic findings in PTTM include multifocal beaded peripheral pulmonary arteries, diffuse thickening of the intralobular septa or peripheral wedge-shaped opacities suggestive of infarction [7]. A VQ scan can reveal peripheral unmatched perfusion defects in patients with tumor emboli [8]. However, this finding is also present in patients with acute pulmonary emboli or CTEPH and cannot be easily distinguished [9]. Pulmonary microvascular cytology has been used as a diagnostic tool to identify circulating tumor cells, however these cells can be mistaken as normal pulmonary megakaryocytes [10]. The presence of right ventricular hypertrophy by cardiac MRI suggested subacute or chronic pulmonary vascular disease in our patient and right and left heart catheterization confirmed the presence of pre-capillary pulmonary hypertension. Therefore other conditions in the differential included drug-induced pulmonary arterial hypertension (PAH) and CTEPH.

The development of hypoxia after the start of prostacyclin treatment raised suspicion for PVOD as the underlying cause of her pulmonary hypertension. Although histopathologically PVOD typically affects post-capillary venules, hemodynamically PVOD has a pre-capillary pattern on right heart catheterization similar to our patient [11]. In addition, the patient’s regimen included multiple chemotherapeutic agents including cisplatin and docetaxel and radiation therapy, all of which are associated with development of PVOD [12, 13]. However, based on the original descriptions of PTTM, concurrent histological evidence of PVOD in our patient was unexpected [1]. Nevertheless, cases of PTTM with pulmonary venous involvement have been previously reported [4, 14].

Many cases of PTTM are treated in a similar manner to idiopathic PAH, although it is unclear such therapies are effective and as demonstrated by this case, they may be harmful. In patients with PVOD, pulmonary edema may develop with pulmonary vasodilator therapy due to increased pulmonary artery blood flow in the face of high post-capillary venule resistance. Alternatively, systemic administration of a potent pulmonary vasodilator may cause ventilation and perfusion mismatching leading to hypoxia. Based on the pulmonary histopathology, the hypoxemia seen in our patient was likely due to both pulmonary edema from venule obstruction and ventilation perfusion mismatch.

In contrast to a suspected role for angiogenic growth factors such as VEGF in the pathogenesis of PTTM, and anecdotal case reports of positive outcomes with imatinib treatment [15] our patient not only developed, but also progressed to symptomatic PTTM during prolonged treatment with a VEGFR inhibitor. Importantly, pharmacokinetic studies done in our patient revealed that co-administration of durvalumab significantly decreased clearance of cediranib [16]. While the effect of VEGFR inhibition on the development or progression of PTTM is unknown, there is experimental evidence that exposure to a single dose of a VEGFR inhibitor, semaxanib (SU-5416), followed by chronic hypoxia leads to angioobliterative PAH in rats that mimics the histopathologic findings in patients with PAH, including hyperproliferative plexiform lesions [17]. In rodents, VEGFR blockade induces widespread pulmonary artery endothelial cell apoptosis which in the presence of chronic hypoxia is thought to result in the development of an apoptosis-resistant, hyperproliferative endothelial cell phenotype [18]. As a result of VEGFR blockade in this model, elevated levels of VEGFR ligands as well as other angiogenic factors (e.g. fibroblast growth factor and placental growth factor) may promote hyperproliferation and vascular remodeling [18]. In patients treated with VEGFR inhibitors, elevated circulating levels of fibroblast growth factor, placental growth factor and hepatocyte growth factor (HGF) have also been detected prior to disease progression [19, 20]. Similarly, in murine models of human non-small cell lung cancer, treatment with vandetanib and cediranib initially led to tumor regression followed by resistance to therapy and progression that was associated with upregulation of both HGF and its receptor, c-MET [21]. Studies of the tumor microvasculature in these murine models revealed HGF-dependent dysregulated angiogenesis with tortuous blood vessel formation. Interestingly, at autopsy immunohistochemical staining revealed lung metastatic foci that were VEGF negative but HGF positive. Thus, it is tempting to speculate that prolonged exposure to high levels of the VEGF receptor inhibitor could similarly provoke an abnormal response in the pulmonary vasculature that either induced or accelerated the development of PTTM in our patient. Moreover, levels of interferon and TNFα as well as other inflammatory cytokines closely linked to pulmonary vascular remodeling in PAH [22–24] may be elevated in the setting of PD-L1 blockade due to compensatory feedback mechanisms [25, 26]. Therefore, although PD1 and PD-L1 inhibitors have not been associated with the development of pulmonary hypertension [27], this ensuing pro-inflammatory state may act synergistically with VEGFR inhibition to disrupt angiogenesis and promote abnormal vessel formation [18]. In our patient, PD-L1 staining was performed on a lymph node taken prior to checkpoint inhibitor therapy and revealed positive staining on approximately 20% of tumor cells. Nevertheless, any association of VEGFR and PD-L1 inhibition to the development of PTTM and PVOD remains speculative.

Histopathology is necessary to definitively diagnose PTTM, yet a surgical lung biopsy is prohibitively risky in the presence of severe pulmonary hypertension and right heart failure. Thus, similar to our report, mechanistic studies of PTTM are lacking due to the difficulty in making a definitive pre-mortem diagnosis. Finally, as a single case report, there are many inherent limitations to our manuscript. The major limitation is the inability to establish causality between the patient’s experimental therapy and her risk for the development of PTTM.

## Conclusion

In the setting of malignancy, PTTM should be included in the differential diagnosis of a patient that presents with subacute to chronic pulmonary hypertension. Serial echocardiography may be useful for identifying evidence of pulmonary hypertension or right ventricular dysfunction prior to the onset of severe symptoms. However, these findings are non-specific and there are no established criteria for screening patients who are at higher risk for developing PTTM. This case report illustrates that a pre-mortem diagnosis of PTTM is difficult to confirm, treatment guidelines are lacking and the prognosis is poor. In addition, our case provides further support for the premise that both PTTM and PVOD share a common pathogenesis and may be manifestations of a spectrum of similar disease processes.

## Abbreviations

CTEPH: chronic thromboembolic pulmonary hypertension
HGF: hepatocyte growth factor
NIH: National Institutes of Health
PAH: pulmonary arterial hypertension
PD-L1: programmed death-ligand 1
PTTM: pulmonary tumor thrombotic microangiopathy
PVOD: pulmonary veno-occlusive disease
TAPSE: tricuspid annular plane systolic excursion
TNFα: tumor necrosis factor alpha
VEGF: vascular endothelial growth factor
VEGFR: vascular endothelial growth factor receptor
VQ scan: ventilation perfusion scan

## References

[1] von Herbay A, Illes A, Waldherr R, Otto HF (1990). Pulmonary tumor thrombotic microangiopathy with pulmonary hypertension. Cancer.

[2] Uruga H, Fujii T, Kurosaki A, Hanada S, Takaya H, Miyamoto A, Morokawa N, Homma S, Kishi K (2013). Pulmonary tumor thrombotic microangiopathy: a clinical analysis of 30 autopsy cases. Intern Med.

[3] Abe H, Hino R, Fukayama M (2013). Platelet-derived growth factor-a and vascular endothelial growth factor-C contribute to the development of pulmonary tumor thrombotic microangiopathy in gastric cancer. Virchows Arch.

[4] Godbole R, Saggar R, Zider A, Betancourt J, Wallace WD, Suh RD, Kamangar N (2017). Insights on pulmonary tumor thrombotic microangiopathy: a seven-patient case series. Pulm Circ.

[5] Gavin MC, Morse D, Partridge AH, Levy BD, Loscalzo J (2012). Clinical problem-solving**. Breathless**. N Engl J Med.

[6] Frazier AA, Franks TJ, Mohammed TL, Ozbudak IH, Galvin JR (2007). From the archives of the AFIP: pulmonary veno-occlusive disease and pulmonary capillary hemangiomatosis. Radiographics.

[7] Restrepo CS, Betancourt SL, Martinez-Jimenez S, Gutierrez FR (2012). Tumors of the pulmonary artery and veins. Semin Ultrasound CT MR.

[8] Boudreau RJ, Lisbona R, Sheldon H (1982). Ventilation-perfusion mismatch in tumor embolism. Clin Nucl Med.

[9] Auger WR, Kerr KM, Kim NH, Fedullo PF (2012). Evaluation of patients with chronic thromboembolic pulmonary hypertension for pulmonary endarterectomy. Pulm Circ.

[10] Masson RG, Krikorian J, Lukl P, Evans GL, McGrath J (1989). Pulmonary microvascular cytology in the diagnosis of lymphangitic carcinomatosis. N Engl J Med.

[11] Rambihar VS, Fallen EL, Cairns JA (1979). Pulmonary veno-occlusive disease: antemortem diagnosis from roentgenographic and hemodynamic findings. Can Med Assoc J.

[12] Ranchoux B, Gunther S, Quarck R, Chaumais MC, Dorfmuller P, Antigny F, Dumas SJ, Raymond N, Lau E, Savale L (2015). Chemotherapy-induced pulmonary hypertension: role of alkylating agents. Am J Pathol.

[13] Kramer MR, Estenne M, Berkman N, Antoine M, de Francquen P, Lipski A, Jacobovitz D, Lafair J (1993). Radiation-induced pulmonary veno-occlusive disease. Chest.

[14] Kumar N, Price LC, Montero MA, Dimopoulos K, Wells AU, Wort SJ (2015). Pulmonary tumour thrombotic microangiopathy: unclassifiable pulmonary hypertension?. Eur Respir J.

[15] Price LC, Wells AU, Wort SJ (2016). Pulmonary tumour thrombotic microangiopathy. Curr Opin Pulm Med.

[16] Lee JM, Cimino-Mathews A, Peer CJ, Zimmer A, Lipkowitz S, Annunziata CM, Cao L, Harrell MI, Swisher EM, Houston N (2017). **Safety and Clinical Activity of the Programmed Death-Ligand 1 Inhibitor Durvalumab in Combination With Poly (ADP-Ribose) Polymerase Inhibitor Olaparib or** Vascular **Endothelial Growth Factor** Receptor **1–3 Inhibitor Cediranib in Women's** Cancers**: A Dose-Escalation, Phase I Study**. J Clin Oncol.

[17] Tuder RM, Chacon M, Alger L, Wang J, Taraseviciene-Stewart L, Kasahara Y, Cool CD, Bishop AE, Geraci M, Semenza GL (2001). Expression of angiogenesis-related molecules in plexiform lesions in severe pulmonary hypertension: evidence for a process of disordered angiogenesis. J Pathol.

[18] Voelkel NF, Gomez-Arroyo J (2014). The role of vascular endothelial growth factor in pulmonary arterial hypertension**. The angiogenesis paradox**. Am J Respir Cell Mol Biol.

[19] Kopetz S, Hoff PM, Morris JS, Wolff RA, Eng C, Glover KY, Adinin R, Overman MJ, Valero V, Wen S (2010). Phase II trial of infusional fluorouracil, irinotecan, and bevacizumab for metastatic colorectal cancer: efficacy and circulating angiogenic biomarkers associated with therapeutic resistance. J Clin Oncol.

[20] Welti J, Loges S, Dimmeler S, Carmeliet P (2013). Recent molecular discoveries in angiogenesis and antiangiogenic therapies in cancer. J Clin Invest.

[21] Cascone T, Xu L, Lin HY, Liu W, Tran HT, Liu Y, Howells K, Haddad V, Hanrahan E, Nilsson MB (2017). The HGF/c-MET pathway is a driver and biomarker of VEGFR-inhibitor resistance and vascular remodeling in non-small cell lung Cancer. Clin Cancer Res.

[22] George PM, Oliver E, Dorfmuller P, Dubois OD, Reed DM, Kirkby NS, Mohamed NA, Perros F, Antigny F, Fadel E (2014). Evidence for the involvement of type I interferon in pulmonary arterial hypertension. Circ Res.

[23] Hurst LA, Dunmore BJ, Long L, Crosby A, Al-Lamki R, Deighton J, Southwood M, Yang X, Nikolic MZ, Herrera B (2017). TNFalpha drives pulmonary arterial hypertension by suppressing the BMP type-II receptor and altering NOTCH signalling. Nat Commun.

[24] Rabinovitch M, Guignabert C, Humbert M, Nicolls MR (2014). Inflammation and immunity in the pathogenesis of pulmonary arterial hypertension. Circ Res.

[25] Kondo A, Yamashita T, Tamura H, Zhao W, Tsuji T, Shimizu M, Shinya E, Takahashi H, Tamada K, Chen L (2010). Interferon-gamma and tumor necrosis factor-alpha induce an immunoinhibitory molecule, B7-H1, via nuclear factor-kappaB activation in blasts in myelodysplastic syndromes. Blood.

[26] Cunningham CR, Champhekar A, Tullius MV, Dillon BJ, Zhen A, de la Fuente JR, Herskovitz J, Elsaesser H, Snell LM, Wilson EB (2016). Type I and type II interferon coordinately regulate suppressive dendritic cell fate and function during viral persistence. PLoS Pathog.

[27] Postow MA, Sidlow R, Hellmann MD (2018). Immune-related adverse events associated with immune checkpoint blockade. N Engl J Med.

